# The mediating role of Body Roundness Index in the association between Life’s Crucial 9 and infertility: a cross-sectional study using NHANES 2013–2018

**DOI:** 10.3389/fnut.2025.1605601

**Published:** 2025-09-16

**Authors:** Ying Wang, Lin Gao, Qianyi Zhong, Lingfen Bao, Jianping Xu, Ling Zhang

**Affiliations:** ^1^Gynecology and obstetrics, Taizhou Central Hospital (Taizhou University Hospital), Taizhou, Zhejiang, China; ^2^Department of Clinical Laboratory, Taizhou Central Hospital (Taizhou University Hospital), Taizhou, Zhejiang, China

**Keywords:** Life’s Crucial 9, Body Roundness Index, infertility, NHANES, mediation analysis

## Abstract

**Background:**

Recent findings indicate a possible connection among heart health, obesity, and infertility. Yet, the processes through which obesity affects the link between heart health and infertility are still not well-understood. The newly created Life’s Crucial 9 (LC9) serves as a measure for evaluating heart health, and the Body Roundness Index (BRI) offers a more accurate and innovative approach to measuring central obesity. The objective of this research is to explore the link between LC9 and infertility and determine if BRI serves as an intermediary in this connection.

**Methods:**

The data for this cross-sectional analysis was sourced from the 2013 to 2018 National Health and Nutrition Examination Survey (NHANES). Following the application of exclusion criteria, 2,319 women aged between 18 and 45 years were incorporated. To investigate the link between LC9, BRI, and infertility, methods like weighted multivariable logistic regression models, restricted cubic spline (RCS) analysis, and subgroup analyses were utilized. Furthermore, an analysis of mediation was performed to determine if BRI played a mediating role in the link between LC9 and infertility.

**Results:**

Within the demographic of the study, infertility occurred in 13% of cases. Post-adjustment for every covariate, a rise of 10 units in LC9 correlated with a 29% decrease in infertility rates (OR = 0.71, 95% CI: 0.61–0.84, *P* < 0.001). In contrast, a one-unit rise in BRI correlated with a 14% increase in infertility rates (OR = 1.14, 95% CI: 1.07–1.23, *P* < 0.001). Analysis using the RCS method revealed a direct negative relationship between LC9 and infertility, and a positive correlation between BRI and infertility. Mediation analysis showed that BRI mediated 16.26% of LC9’s overall impact on infertility (*P* < 0.001), suggesting a substantial influence of central obesity in this correlation.

**Conclusion:**

There is a significant negative correlation between LC9 and infertility, with BRI playing a partial mediating role. These findings highlight the importance of cardiovascular health and obesity management in reproductive health and suggest that reducing central obesity may lower the risk of infertility. Further research is needed on potential intervention strategies targeting metabolic and cardiovascular health to prevent infertility.

## Introduction

Infertility is a significant public health concern, affecting an estimated 10%–15% of couples worldwide (1, 2). The condition is characterized by the inability to conceive after 12 months of regular, unprotected intercourse, imposing substantial psychological, social, and economic burdens on affected individuals and society (3). The prevalence of infertility has been rising, potentially due to a combination of environmental, lifestyle, and metabolic factors (4). Despite advancements in assisted reproductive technologies, infertility remains a challenging condition with limited treatment success rates (5, 6). Identifying modifiable risk factors that contribute to infertility is crucial for developing preventive strategies, ultimately reducing its burden and improving reproductive health outcomes.

Previous studies have suggested a complex interplay between infertility and cardiovascular health (7, 8). Emerging evidence indicates that individuals with infertility, particularly women, may have an increased risk of developing cardiovascular diseases later in life (9). This connection underscores the importance of evaluating lifestyle and cardiometabolic factors in the context of infertility. Life’s Crucial 9 (LC9), a composite measure of cardiovascular and metabolic health developed by the American Heart Association, encompasses key lifestyle and biological factors such as diet, physical activity, blood glucose, and body mass index (10). Given the shared metabolic pathways between cardiovascular disease and reproductive dysfunction, exploring the relationship between LC9 and infertility could provide novel insights into potential prevention strategies. However, the extent to which LC9 is associated with infertility remains underexplored, necessitating further investigation.

Obesity has long been recognized as a key contributor to both cardiovascular conditions and reproductive dysfunction, primarily through its effects on endocrine balance, chronic inflammation, and metabolic disturbances (11, 12). Conventional measures like body mass index (BMI), though widely used, fall short in capturing fat distribution patterns and do not fully reflect the associated metabolic risks (13). The Body Roundness Index (BRI), a novel obesity metric, incorporates both waist circumference and height to better capture central adiposity and its associated health risks (14, 15). Given that obesity plays a critical role in both cardiovascular health and infertility, examining whether BRI mediates the relationship between LC9 and infertility could provide mechanistic insights into obesity’s contribution to reproductive dysfunction. The National Health and Nutrition Examination Survey (NHANES) 2013–2018 cycles offer a unique opportunity to investigate this relationship in a large, nationally representative sample. NHANES provides comprehensive health and lifestyle data, allowing for robust statistical analyses to elucidate the mediating role of BRI in the LC9-infertility association. Understanding these interrelationships may inform targeted interventions to improve reproductive and cardiometabolic health outcomes.

## Materials and methods

### Study participants

We conducted a cross-sectional study utilizing data collected from the 2013 to 2018 cycles of the National Health and Nutrition Examination Survey (NHANES). This nationwide program, administered by the National Center for Health Statistics (NCHS), is designed to evaluate the health and dietary patterns of the United States population using a stratified, multistage sampling approach to achieve demographic representativeness. Ethical oversight was provided by the NCHS Institutional Review Board, and informed consent was obtained from all participants prior to data collection. Since this study was a secondary data analysis based on a public database and did not involve personal privacy information, no additional ethical review approval was required in accordance with the STROBE guidelines for cross-sectional studies.

We initially screened 29,400 participants from the 2013 to 2018 NHANES dataset. Individuals who did not meet the age range (18–45 years) or were male were subsequently excluded (*n* = 25,077). Of the remaining 4,323 women, participants with incomplete LC9 information (*n* = 1,973) and individuals with infertility or missing BRI data (*n* = 31) were further excluded. Finally, a total of 2,319 eligible participants were included in the analysis of this study (see Supplementary Figure 1 for details).

### Definition of Life’s Crucial 9

Life’s Crucial 9 serves as an integrative metric designed to evaluate overall cardiometabolic wellbeing, encompassing nine core elements. These include four lifestyle-related behaviors—dietary habits, physical exercise, tobacco exposure, and sleep patterns—and five physiological indicators: body weight, arterial pressure, glucose regulation, lipid levels, and psychological status. Each dimension is quantified based on standardized procedures outlined in the NHANES framework, with scoring criteria aligned with contemporary cardiovascular health recommendations.

For each participant, the LC9 total score is obtained by averaging the scores of these nine components, and the score range of each indicator is 0–100. Among them, dietary quality is assessed based on the Healthy Eating Index-2015 (HEI-2015), and the detailed scoring method is listed in Supplementary Table 2. Information on physical activity, tobacco use, and sleep quality is collected via standardized self-report questionnaires. In contrast, indicators such as BMI, blood pressure, glucose levels, and serum lipids are measured directly by qualified healthcare professionals using clinical and laboratory-based procedures.

Mental health status was assessed using the Patient Health Questionnaire-9 (PHQ-9), which reflects the severity of depressive symptoms through different score intervals. The specific scoring criteria and quantification methods for each LC9 component are detailed in Supplementary Table 1.

### Definition of BRI and infertility

The BRI is a new anthropometric index that aims to more accurately reflect the distribution of body fat, especially central obesity, compared with the traditional BMI. BRI combines waist circumference and height as two key parameters to more effectively assess the degree of abdominal obesity and the health risks it brings, such as cardiovascular disease and metabolic disorders. The calculation of BRI is based on a standardized formula that can provide a quantitative estimate of individual fat distribution.

Regarding the identification of infertility, this study determined the infertility status of participants based on two key questions in the NHANES Reproductive Health Questionnaire. The two questions are:

RHQ074: “Have you tried to get pregnant for more than 1 year but failed?”

RHQ076: “Have you ever visited a doctor or other medical institution because of difficulty in getting pregnant?”

If the participant answered “yes” to any of the above questions, they were classified as “having a history of infertility” and included in the infertility group; if they answered “no” to both questions, they were considered to have no infertility experience and were classified as the non-infertility group.

### Covariates

This study accounted for a range of covariates, encompassing sociodemographic variables—such as age, ethnicity, marital condition, educational attainment, and the poverty-to-income ratio (PIR)—as well as key clinical indicators, including the presence of hypertension, diabetes mellitus, and elevated blood lipid levels. Age was divided into three groups: 18–25, 26–34, and 35–45 years. Participants were categorized by race into groups such as Mexican American, Black (non-Hispanic), White (non-Hispanic), and a collective group of other ethnicities. Marital classification distinguished individuals as either “married” or “unmarried,” based on whether they were in a legal or cohabiting partnership. The PIR index was used to assess socioeconomic status, where PIR < 1.3 indicated poverty.

The definition of clinical covariates followed the NHANES criteria. Diabetes was determined based on self-reported history of diabetes, glycated hemoglobin (HbA1c) level ≥ 6.5%, or fasting blood glucose ≥ 126 mg/dL. Hypertension was defined as a self-reported history of hypertension, taking antihypertensive medication, systolic blood pressure (SBP) ≥ 140 mmHg, or diastolic blood pressure (DBP) ≥ 90 mmHg. The diagnostic criteria for hyperlipidemia included triglycerides (TG) ≥ 150 mg/dL, total cholesterol (TC) ≥ 200 mg/dL, low-density lipoprotein (LDL) ≥ 130 mg/dL, or high-density lipoprotein (HDL) < 40 mg/dL (male) or < 50 mg/dL (female); in addition, individuals taking lipid-lowering drugs were also considered to have hyperlipidemia. The specific definitions and classifications of all covariates are detailed in Supplementary Table 3.

### Statistical analysis

All statistical computations were performed with R (v4.3.1), applying the designated NHANES weighting scheme to derive population-representative outcomes. The weighting variable “WTMEC2YR” was used, and for the 2013–2018 NHANES cycles, a new weight was calculated as 1/3 × WTMEC2YR to account for the combined survey periods. Continuous variables were presented as mean ± standard deviation (SD), with weighted *t*-tests used to compare differences between groups. Categorical variables were expressed as weighted percentages (*N*, %), and group differences were assessed using weighted chi-square tests. To explore the links among LC9, BRI, and infertility, we utilized multivariate logistic regression with a three-tier modeling approach:

1. Model 1: Baseline analysis without covariate adjustment.
2. Model 2: Controlled for age, marital status, educational background, income level (PIR), and ethnicity.
3. Model 3: Further adjusted for clinical comorbidities including hypertension, diabetes, and dyslipidemia.

To capture potential non-linear associations, a restricted cubic spline (RCS) approach was employed. Stratified analyses were also carried out to detect heterogeneity across subpopulations defined by demographic and clinical parameters. Furthermore, mediation analysis was conducted to assess the extent to which the effect of LC9 on infertility was mediated by BRI. We used a non-parametric bootstrap resampling procedure with 5,000 iterations to estimate the indirect, direct, and total effects (16). The indirect effect was defined as the product of the regression coefficient from LC9 to BRI (Path A) and from BRI to infertility (Path B), while the direct effect (Path C’) represented the association between LC9 and infertility after adjusting for BRI. The total effect (Path C) was calculated as the sum of direct and indirect effects. The proportion of mediation was computed as: (indirect effect/total effect) × 100. All mediation models were implemented using the R package “mediation,” and statistical significance was determined based on bootstrap-derived confidence intervals and two-tailed *P*-values < 0.05.

## Results

### Baseline characteristics

This study ultimately enrolled 2,319 individuals who met the inclusion criteria, representing approximately 26.15 million United States adults. The overall prevalence of infertility is 13%, equivalent to approximately 3.27 million people affected. Compared with individuals without infertility, infertile patients had significant differences in multiple aspects, including age, marital status, the prevalence of hypertension, diabetes, hyperlipidemia, LC9 scores, and BRI levels (all *P* < 0.05).

Specifically, the average age of participants in the infertile group was older, the prevalence of cardiovascular and metabolic diseases (such as hypertension, diabetes, and hyperlipidemia) was higher, and the LC9 score was significantly lower, indicating that their overall health behaviors and physiological status were poor. In addition, the BRI level was significantly increased in the infertile group, and the proportion of individuals with high BRI (the highest tertile group) was also higher, reflecting the higher concentration of central obesity in the infertile population. These results suggest that cardiovascular and metabolic health status may play an vital role in the occurrence of infertility. Detailed data on relevant baseline characteristics are shown in Table 1.

**TABLE 1:** Weighted baseline characteristics stratified by infertility status.

Characteristic	Overall, *N* = 26,147,035 (100%)	Non-infertility, *N* = 22,872,649 (87%)	Infertility, *N* = 3,274,386 (13%)	*P*-value
No. of participants in the sample	2,319	2,043	276	–
**Age (%)**				**< 0.001**
18–25	6,219,351 (24%)	5,845,220 (26%)	374,131 (11%)	–
26–34	8,707,347 (33%)	7,714,940 (34%)	992,407 (30%)	–
35–45	11,220,338 (43%)	9,312,490 (41%)	1,907,848 (58%)	–
**Race (%)**				0.428
Non-Hispanic White	14,819,649 (57%)	12,817,421 (56%)	2,002,228 (61%)	–
Other	4,752,502 (18%)	4,275,874 (19%)	476,628 (15%)	–
Non-Hispanic Black	3,402,249 (13%)	2,998,983 (13%)	403,266 (12%)	–
Mexican American	3,172,635 (12%)	2,780,371 (12%)	392,263 (12%)	–
**Married/live with partner (%)**				**< 0.001**
No	10,578,918 (40%)	9,835,008 (43%)	743,910 (23%)	–
Yes	15,568,117 (60%)	13,037,641 (57%)	2,530,475 (77%)	–
**Education level (%)**				0.731
Below high school	2,783,558 (11%)	2,415,019 (11%)	368,539 (11%)	–
High School or above	23,363,477 (89%)	20,457,630 (89%)	2,905,847 (89%)	–
**PIR (%)**				0.088
Poor	6,998,474 (29%)	6,228,667 (29%)	769,807 (24%)	–
Not Poor	17,524,388 (71%)	15,105,830 (71%)	2,418,558 (76%)	–
**Hypertension (%)**				**0.001**
No	22,343,080 (85%)	19,772,606 (86%)	2,570,473 (79%)	–
Yes	3,803,955 (15%)	3,100,043 (14%)	703,912 (21%)	–
**Diabetes (%)**				**0.005**
No	24,877,400 (95%)	21,890,152 (96%)	2,987,249 (91%)	–
Yes	1,269,635 (4.9%)	982,498 (4.3%)	287,137 (8.8%)	–
**Hyperlipidemia (%)**				**0.020**
No	12,294,565 (47%)	11,043,683 (48%)	1,250,881 (38%)	–
Yes	13,852,470 (53%)	11,828,966 (52%)	2,023,504 (62%)	–
Mean LC9 score [mean (SD)]	76.03 (13.52)	76.78 (13.27)	70.78 (14.13)	**< 0.001**
**LC9, tertile (%)**				**< 0.001**
T1	8,636,800 (33%)	7,121,334 (31%)	1,515,466 (46%)	–
T2	8,604,254 (33%)	7,517,443 (33%)	1,086,810 (33%)	–
T3	8,905,982 (34%)	8,233,872 (36%)	672,110 (21%)	–
Mean psychological health score [mean (SD)]	86.71 (25.81)	87.42 (25.01)	81.71 (30.43)	**0.034**
Mean HEI-2015 diet score [mean (SD)]	38.21 (31.73)	38.69 (31.96)	34.88 (29.87)	0.177
Mean physical activity score [mean (SD)]	77.59 (38.77)	78.14 (38.34)	73.74 (41.50)	0.170
Mean tobacco exposure score [mean (SD)]	75.50 (39.10)	76.37 (38.54)	69.39 (42.35)	**0.028**
Mean sleep health score [mean (SD)]	84.62 (23.25)	85.06 (22.96)	81.48 (25.00)	0.086
Mean body mass index score [mean (SD)]	60.02 (36.97)	61.67 (36.42)	48.46 (38.72)	**0.001**
Mean blood lipid score [mean (SD)]	80.21 (26.32)	80.87 (25.89)	75.59 (28.77)	**0.041**
Mean blood glucose score [mean (SD)]	93.86 (16.60)	94.51 (15.82)	89.31 (20.76)	**< 0.001**
Mean blood pressure score [mean (SD)]	87.52 (22.05)	88.24 (21.58)	82.44 (24.52)	**0.005**
BRI [mean (SD)]	5.40 (2.70)	5.25 (2.60)	6.47 (3.14)	**< 0.001**
**BRI, tertile (%)**				**< 0.001**
T1	8,719,893 (33%)	8,069,353 (35%)	650,540 (20%)	–
T2	8,714,472 (33%)	7,699,087 (34%)	1,015,385 (31%)	–
T3	8,712,670 (33%)	7,104,209 (31%)	1,608,460 (49%)	–

Continuous variables are expressed as mean (standard deviation), with *P*-values derived from weighted Student’s *t*-tests. Categorical variables are reported as weighted counts and percentages (*N*, %), and their group differences were assessed using the weighted chi-square test. LC9, Life’s Crucial 9; BRI, Body Roundness Index; PIR, poverty-income ratio. Bold values indicate *p* < 0.05.

### Association between LC9, BRI, and infertility

As illustrated in Table 2, a trio of logistic regression models was applied to examine the link between LC9, BRI, and infertility. Across all models, LC9 consistently showed a statistically significant inverse association with infertility risk (*P* < 0.001). Specifically, in Model 3—which controlled for variables including age, educational background, marital status, poverty-income ratio (PIR), ethnicity, hypertension, diabetes, and hyperlipidemia—each 10-point increment in LC9 corresponded to a 29% decrease in infertility prevalence [odds ratio (OR): 0.71, 95% confidence interval (CI): 0.61–0.84].

**TABLE 2:** Association between LC9, BRI, and infertility.

Characteristics	Model 1 [OR (95% CI)]	*P*-value	Model 2 [OR (95% CI)]	*P*-value	Model 3 [OR (95% CI)]	*P*-value
**LC9 - infertility**						
Continuous (per 10 scores)	0.73 (0.64,0.84)	< 0.001	0.71 (0.62, 0.80)	< 0.001	0.71 (0.61, 0.84)	< 0.001
**Tertile**						
T1	1 (ref.)	–	1 (ref.)	–	1 (ref.)	–
T2	0.68 (0.47, 0.98)	0.040	0.64 (0.44, 0.93)	0.020	0.68 (0.45, 1.02)	0.060
T3	0.38 (0.23, 0.63)	< 0.001	0.36 (0.22, 0.59)	< 0.001	0.39 (0.22, 0.69)	0.002
*P* for trend	< 0.001		<0.001		0.002	
**BRI - infertility**						
Continuous	1.15 (1.08, 1.23)	< 0.001	1.16 (1.08, 1.23)	< 0.001	1.14 (1.07, 1.23)	< 0.001
**Tertile**						
T1	1 (ref.)	–	1 (ref.)	–	1 (ref.)	–
T2	1.64 (1.06, 2.51)	0.030	1.60 (0.97, 2.63)	0.060	1.55 (0.93, 2.59)	0.090
T3	2.81 (1.72, 4.60)	< 0.001	2.85 (1.66, 4.92)	< 0.001	2.64 (1.49, 4.69)	0.002
*P* for trend	< 0.001		< 0.001		0.001	

Model 1: unadjusted, with no confounding variables included. Model 2: adjusted for demographic and socioeconomic factors—age, educational attainment, marital status, PIR, and ethnicity. Model 3: further incorporated clinical covariates, including hypertension, diabetes, and dyslipidemia, in addition to Model 2 variables. LC9, Life’s Crucial 9; BRI, Body Roundness Index; PIR, poverty-income ratio; OR, odds ratio; CI, confidence interval.

When stratified by tertiles, individuals falling into the highest LC9 group (T3) demonstrated a 61% lower infertility rate compared to those in the lowest category (T1) [OR: 0.39, 95% CI: 0.22–0.69, *P* = 0.002].

Similarly, BRI was positively associated with infertility in all models (*P* < 0.001). In Model 3, each unit increase in BRI was associated with a 14% higher infertility prevalence [OR: 1.14, 95% CI: 1.07–1.23]. When analyzed by tertiles, participants in the highest BRI tertile (T3) had a 2.64-fold higher prevalence of infertility compared to those in the lowest tertile (T1) [OR: 2.64, 95% CI: 1.49–4.69, *P* = 0.002], demonstrating a significant trend.

**FIGURE 1:**
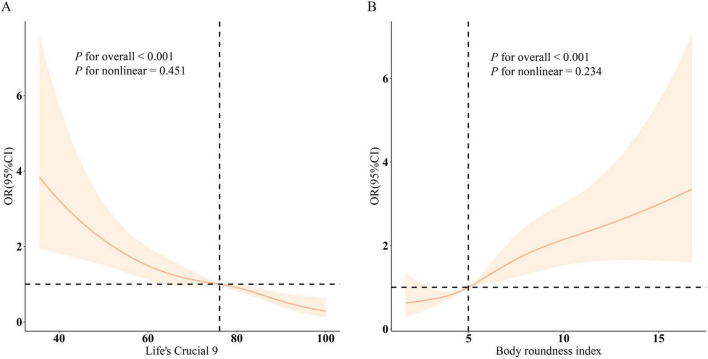
Dose–response curves depicting the associations between Life’s Crucial 9 (LC9), Body Roundness Index (BRI), and infertility risk. **(A)** shows the relationship between LC9 and infertility, while **(B)** illustrates the association for BRI. Odds ratios (solid lines) with corresponding 95% confidence intervals (shaded regions) were estimated after adjusting for age, marital status, educational level, poverty-to-income ratio (PIR), race, and cardiometabolic comorbidities including hypertension, diabetes, and hyperlipidemia.

**FIGURE 2:**
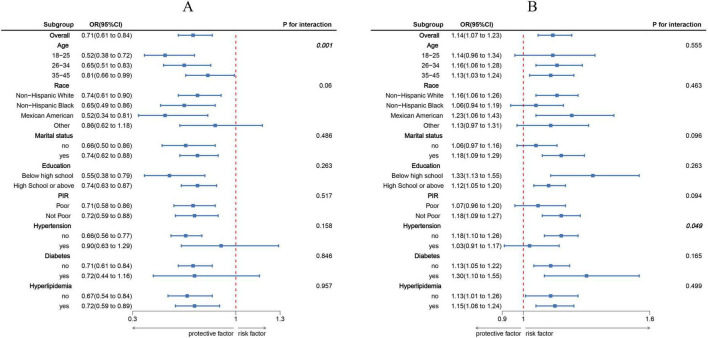
Subgroup analyses exploring the associations of Life’s Crucial 9 (LC9) and Body Roundness Index (BRI) with infertility across various populations. **(A)** illustrates the effect of LC9 (per 10-point increment), while **(B)** presents the impact of BRI (per standard deviation increase). All estimates were derived from models adjusted for age, marital status, educational attainment, poverty-to-income ratio (PIR), race/ethnicity, and comorbid conditions including hypertension, diabetes, and dyslipidemia.

Findings from the RCS analysis (Figure 1) demonstrated a clear dose-response association involving LC9, BRI, and infertility. A negative linear trend was detected between LC9 scores and infertility rates, whereas BRI showed a positive correlation with infertility prevalence. Stratified analyses based on variables such as age, ethnicity, marital status, educational attainment, PIR, and cardiometabolic status (Figure 2) validated these trends across the majority of subgroups. Notably, a significant interaction effect emerged between LC9 and age (*P* < 0.05), indicating that the influence of cardiometabolic health on infertility outcomes may differ depending on age group. No meaningful interactions were identified for other factors, including educational level and PIR.

### Mediation effect

**FIGURE 3:**
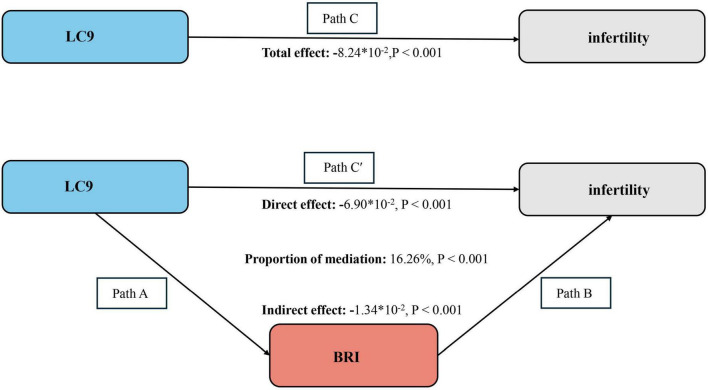
Bootstrap-based mediation framework showing BRI’s indirect effect between LC9 and infertility. Path C represents the overall effect, while path C′ corresponds to the direct effect. The indirect effect is derived by multiplying paths A and B (A × B). The proportion mediated is calculated as: indirect effect ÷ (indirect effect + direct effect) × 100%. LC9, Life’s Crucial 9; BRI, Body Roundness Index. All models were adjusted for age, marital status, education level, poverty-income ratio (PIR), race, hypertension, diabetes, and dyslipidemia.

The mediation model is illustrated in Figure 3, with LC9 as the independent variable, infertility as the dependent variable, and BRI as the mediator. As shown in Supplementary Table 4, a significant association was observed between LC9 and BRI after adjusting for all covariates (β = −1.00, 95% CI: −1.10, −0.93, *P* < 0.001), indicating that lower LC9 scores were associated with higher BRI levels.

The mediation analysis further confirmed the indirect effect of BRI in the association between LC9 and infertility. As shown in the Figure 3, the total effect of LC9 on infertility was significant (−8.24 × 10^–2^, *P* < 0.001). Following the adjustment for all covariates, the direct effect of LC9 on infertility remained statistically significant (β = −6.90 × 10^–2^, *P* < 0.001), while the indirect effect mediated by BRI was also significant (β = −1.34 × 10^–2^, *P* < 0.001). The proportion of the total effect mediated by BRI was estimated to be 16.26% (*P* < 0.001), indicating that BRI partially mediates the relationship between LC9 and infertility. These findings suggest that obesity, as captured by BRI, plays a crucial role in the pathway linking cardiovascular health with infertility risk.

## Discussion

This analysis included data from 2,319 women enrolled in the 2013–2018 NHANES cycles. The results indicated an inverse relationship between LC9 scores and infertility risk, whereas BRI showed a direct positive correlation with infertility. Further mediation analysis revealed that BRI served as a partial mediator in the LC9–infertility pathway, explaining approximately 16.26% of the total effect. These findings highlight the critical role of cardiovascular and metabolic health in reproductive function and suggest that obesity, particularly central adiposity, may be a key pathway linking poor cardiovascular health to infertility.

To our knowledge, this is the first study to examine the relationship between LC9 and infertility with BRI as a mediator. Previous studies have linked cardiovascular health and metabolic dysfunction with reproductive health outcomes, with obesity being recognized as a significant contributor to anovulation, hormonal imbalances, and systemic inflammation (17–20). Our findings are consistent with prior research demonstrating that poor cardiovascular health, as measured by Life’s Simple 7 (LS7) or other cardiovascular health indices, is associated with reduced fertility (21, 22). Similarly, BRI has been shown to be a strong predictor of metabolic syndrome and obesity-related infertility (23, 24), aligning with our results that indicate higher BRI is significantly associated with increased infertility prevalence. However, previous studies have largely focused on BMI-based assessments of obesity (25), which do not accurately reflect body fat distribution and metabolic risk. By incorporating BRI, our study provides a more precise evaluation of central obesity’s impact on infertility.

### Potential mechanisms linking LC9, BRI, and infertility

The components of LC9 influence reproductive health through several biological mechanisms. Dietary quality, physical activity, and metabolic control are crucial factors in maintaining optimal hormonal balance and ovulatory function (26). Poor dietary habits, particularly excessive intake of ultra-processed foods and refined carbohydrates, contribute to systemic inflammation and insulin resistance, which are closely linked to polycystic ovary syndrome (PCOS), anovulation, and impaired endometrial receptivity (27, 28). Conversely, adherence to a heart-healthy diet rich in fiber, antioxidants, and essential micronutrients can improve metabolic function and reproductive outcomes (29, 30).

Physical activity, another critical component of LC9, influences infertility through multiple pathways. Regular moderate-to-vigorous exercise improves insulin sensitivity, reduces systemic inflammation, and regulates reproductive hormone levels, particularly androgens and estrogen, thereby promoting ovulatory function (31, 32). Additionally, increased physical activity reduces abdominal fat accumulation, which is a major contributor to hormonal imbalances and infertility risk (33).

Obesity, particularly central adiposity as measured by BRI, plays a crucial role in reproductive dysfunction. The accumulation of visceral adipose tissue (VAT) is associated with low-grade chronic inflammation, increased oxidative stress, and elevated levels of inflammatory cytokines (e.g., IL-6, TNF-α, CRP), all of which negatively impact ovarian reserve and endometrial receptivity (34–36). VAT is also strongly linked to insulin resistance and hyperinsulinemia, which can disrupt hypothalamic-pituitary-ovarian (HPO) axis function, leading to anovulation and subfertility (37). Our mediation analysis suggests that central obesity, as reflected by BRI, is a significant intermediary in the association between LC9 and infertility, highlighting the importance of obesity management in reproductive health interventions.

### Subgroup findings and implications

Subgroup analyses revealed a significant interaction between LC9 and age, indicating that the protective effects of LC9 on infertility were more evident in women aged 26–45 years, while the association was weaker and statistically non-significant in the youngest age group (18–25 years), potentially due to limited cases of infertility. Aging is a well-established risk factor for diminished ovarian reserve and reduced fertility, and poor cardiovascular health may further exacerbate this decline (38, 39). Future longitudinal studies are warranted to investigate whether improving LC9 components in early adulthood could delay reproductive aging and enhance fertility outcomes.

This study has several strengths. First, it is the first large-scale, population-based study to examine the association between LC9, BRI, and infertility using nationally representative NHANES data, enhancing the generalizability of our findings. Second, the use of BRI instead of traditional BMI measures provides a more accurate assessment of body fat distribution, addressing limitations of prior obesity-related infertility studies. Third, our study employs multiple statistical approaches, including multivariable logistic regression, RCS analysis, and mediation analysis, allowing for a comprehensive evaluation of the direct and indirect effects of LC9 on infertility.

However, several limitations should be acknowledged. First, the cross-sectional design of NHANES prevents us from establishing causality between LC9, BRI, and infertility. Future prospective cohort studies are needed to confirm whether improving cardiovascular health and reducing central adiposity can directly enhance fertility outcomes. Second, infertility in this study was assessed through self-reported responses to two NHANES questionnaire items, which may introduce recall bias and potential misclassification. Specifically, because the survey did not differentiate between primary and secondary infertility, participants who experienced either condition were grouped together. This lack of distinction may lead to heterogeneity within the infertility group and could influence the observed associations. Future studies should incorporate more detailed clinical assessments or validated diagnostic criteria to distinguish between different infertility subtypes, thereby improving the accuracy and interpretability of findings. Third, while we adjusted for major confounders, unmeasured variables, such as genetic predisposition, environmental exposures, and psychosocial stress, may also contribute to infertility risk and were not accounted for in this analysis. Fourth, the study population was limited to women aged 18–45 years, and our findings may not be generalizable to adolescent or postmenopausal populations.

## Conclusion

This study provides evidence that better cardiovascular health, as measured by LC9, is associated with a lower risk of infertility, while higher central adiposity, captured by BRI, is linked to an increased risk of infertility. Furthermore, BRI was found to partially mediate the association between LC9 and infertility, suggesting that obesity, particularly excess abdominal fat, plays a significant role in the relationship between cardiovascular health and reproductive function. These findings highlight the need for a broader, integrative approach to infertility prevention, focusing not only on reproductive health but also on metabolic and cardiovascular wellbeing.

Given the cross-sectional design of this study, future longitudinal research is necessary to confirm these associations and explore whether interventions targeting cardiovascular health and obesity management can improve fertility outcomes. Additionally, further studies should investigate other potential mediators and confounders, such as inflammatory markers, hormonal profiles, and mental health factors, to gain a more comprehensive understanding of the mechanisms underlying the LC9-infertility relationship. Addressing these factors may contribute to more effective public health strategies and clinical interventions aimed at improving both cardiovascular and reproductive health.

## Data Availability

The original contributions presented in this study are included in this article/Supplementary material, further inquiries can be directed to the corresponding authors.
